# Feasibility of peroral endoscopic myotomy with a disposable endoscope platform

**DOI:** 10.1016/j.vgie.2023.10.001

**Published:** 2023-10-10

**Authors:** Tara Keihanian, Salmaan A. Jawaid, Mohamed O. Othman

**Affiliations:** Division of Gastroenterology, Department of Medicine, Baylor College of Medicine, Houston, Texas

## Abstract

Video 1 Demonstration of a successful peroral endoscopic myotomy using a novel disposable scope platform.

Demonstration of a successful peroral endoscopic myotomy using a novel disposable scope platform.

## Case Description

Peroral endoscopic myotomy (POEM) is an established therapeutic modality for achalasia. POEM requires precise movements during each step of the procedure, including tunneling and myotomy. The procedure is long and uses a heavy scope weight, so it can be cumbersome with accumulating ergonomic effects over time. In this case, we describe a successful POEM procedure using a novel disposable gastroscope. Our patient is a 73-year-old man with type III achalasia and prior botulinum toxin injection with temporary response. Informed consent was obtained.

## Procedure

**Figure 1:**
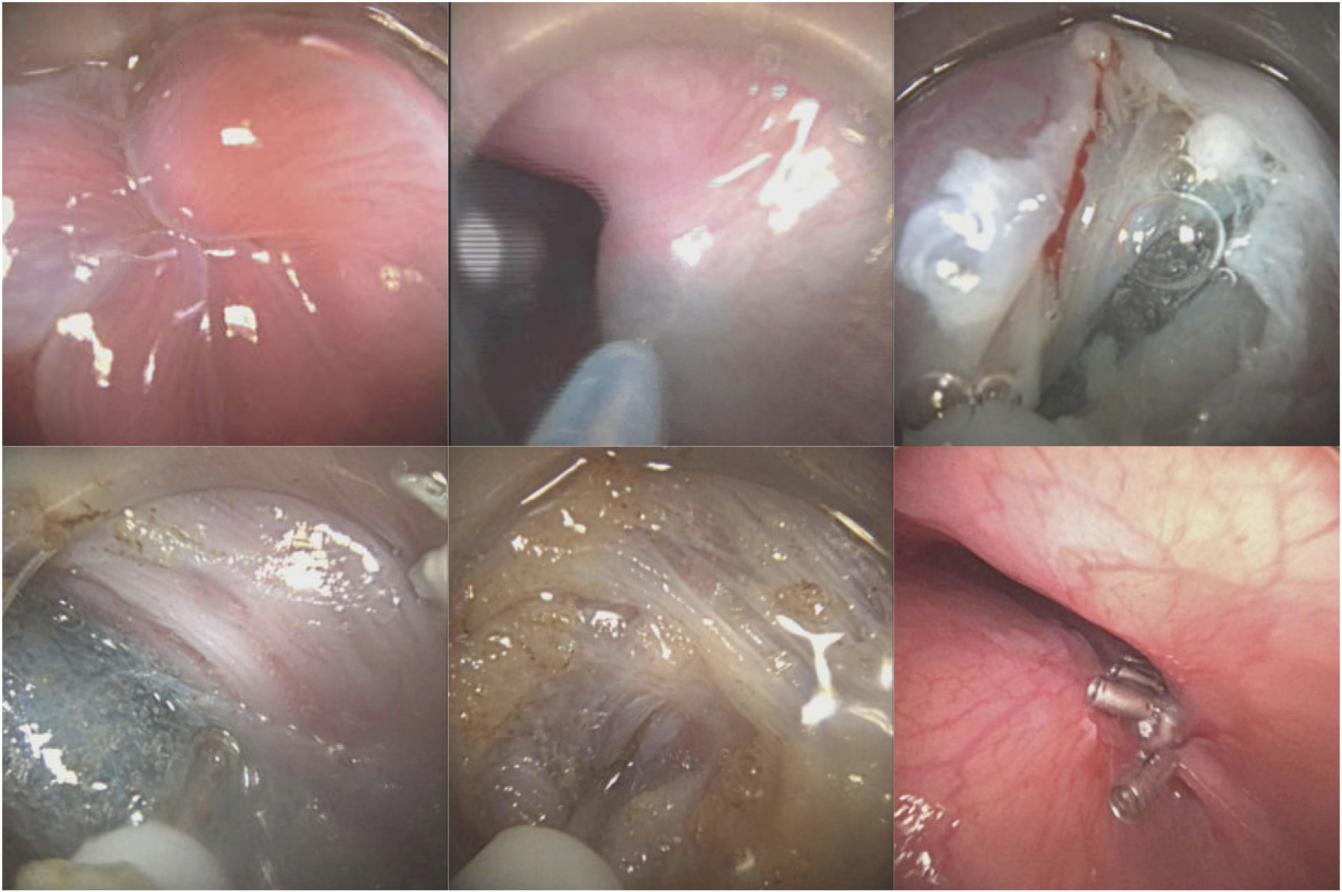
Step-by-step demonstration of peroral endoscopic myotomy with a novel single-use endoscopic platform.

A novel disposable endoscope (AMBU single-use disposable gastroscope; AMBU USA, Columbia, Md, USA) was advanced into the stomach. A hypertonic lower esophageal sphincter was found with moderate resistance passing the scope into the stomach (Fig. 1A). A mixture of methylene blue and saline was injected to create a submucosal cushion 12 cm above the gastroesophageal junction (GEJ) at 2 o’clock using an injection needle (NeedleMaster; Olympus America Inc, Center Valley, Pa, USA) (Fig. 1B). A 2-cm longitudinal incision was then made into the submucosa using an electrocautery dissection knife (Hybrid T knife; Erbe USA, Inc, Marietta, Ga, USA) with an ERBE generator (Erbe USA, Inc) using ENDO CUT Q mode (effect 2, cutting duration 3, cutting interval 3) (Fig. 1C). The endoscope with a cap was advanced into the tunnel, and the submucosal tunnel was created using the electrocautery knife and a series of submucosal injections using “Precise Sect” mode (Precise Sect, 5.7) (Fig. 1D). The tunnel was stopped in the lesser curvature of the stomach, 2 cm below the GEJ. Subsequently, a full-thickness myotomy was performed (Fig. 1E). The initial incision was then closed with 6 hemostatic clips (Dura Clips, 11mm; ConMed USA, Utica, NY, USA) (Fig. 1F). At the conclusion of the procedure, we were able to advance the endoscope through the GEJ without any resistance (Video 1, available at www.videogie.org). An esophagram on the following day did not reveal any leak, and he was discharged without any adverse events. His Eckert score improved to 0 from 5 at his 4-week follow-up. A follow-up endoscopy at 6 months showed a widely patent GEJ.

## Conclusion

**Figure 2:**
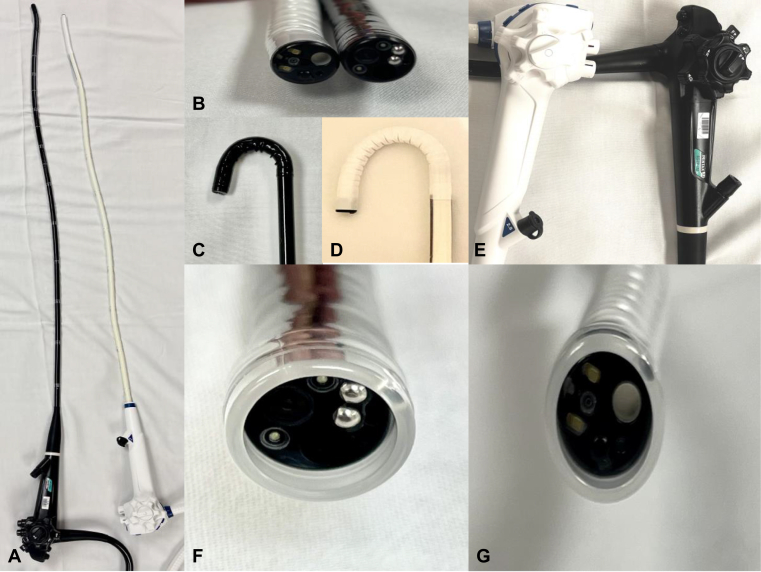
Side-by-side ex vivo comparison of disposable scope and reusable scope (**A,** length, **B**. distal tip width/design, **C** and **D,** retroflexion capability, **E,** scope handle, and **F** and **G,** compatibility with distal clear attachment cap).

**Figure 3:**
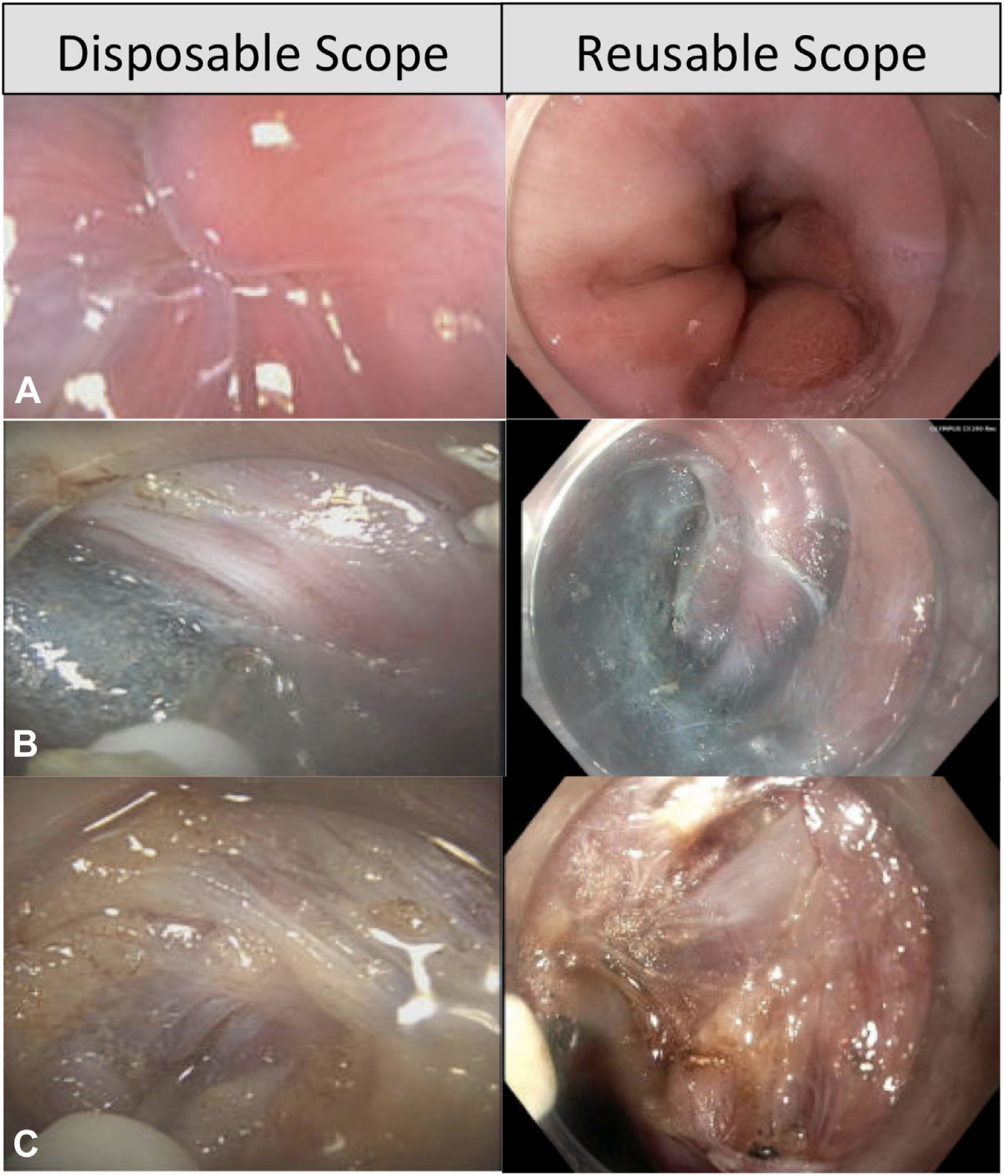
Side-by-side demonstration of the image quality by a disposable scope and a reusable scope (**A,** forward scope viewing, **B,** tunneling, and **C,** myotomy).

In comparison to reusable endoscopes, a disposable scope has a similar length (1030 mm), provides the same degree of retroflexion (up 210°, down 90°, right 100°, left 100°), and is able to fit with the clear attachment caps (Fig. 2, Table 1). Despite the lack of high-definition electrochromoendoscopy optics, the disposable endoscope provided adequate-quality endoscopic illumination during all stages of POEM, including submucosal injection, incision/tunnel entry, dissection, myotomy, closure, and retroflexion view within the stomach (Fig. 3). Despite the lower pixel density of the disposable endoscope, the color contrast between the submucosa and the muscle layer was satisfactory to enable safe and efficient tunneling. During myotomy, it clearly differentiated the circular and longitudinal muscles with favorable image quality (Fig. 3). The 2.8-mm inner working channel allows for easy passage of endoscopic equipment such as an electrocautery knife and hemostatic clips with adequate and satisfactory rotatability. Despite its flexibility, the device has sufficient stiffness for quick tunnel entry, similar to the reusable endoscope. Considering the similarities in design of the disposable endoscope with the reusable endoscope, we anticipate no learning curve associated with this device. Disposable endoscopes are not only suitable for use in immunocompromised patients and those with multidrug-resistant organisms, but also because of their flexible design and lighter weight, which is advantageous ergonomically for longer, complex procedures such as POEM to prevent long-term muscle injury. The future design of disposable endoscopic platforms should be tailored to advance specific needs of each complex therapeutic procedure and to address current limitations of this platform, such as lack of image enhancement and near-focus capabilities.

**Table 1:** Side-by-side comparison of reusable and disposable scopes

	Reusable scope	Disposable scope
Working length	1030 mm	1030 mm
Distal end diameter	9.9 mm	9.9 mm
Working channel inner diameter	2.8 mm	2.8 mm
Bending angulation	Up 210°	Up 210°
	Down 90°	Down 90°
	Right 100°	Right 100°
	Left 100°	Left 100°
Optical system		
Field of view	Normal 140°	Normal 140°
Direction of view	Forward viewing	Forward viewing
Depth of field	5-100 mm	3-100 mm
High-definition format, pixels	1280 × 1024	1080 × 1080
Image enhancement	Narrow-band imaging, near focus	Advanced red contrast∗
Weight	3900 g	650 g

∗ Illumination intensity is proprietary.

## Disclosure

Dr Othman is a consultant for Olympus, Boston Scientific Corporation, AbbVie, ConMed, Lumendi, AMBU, Creo Medical, Neptune Medical, and Apollo; he has also received research grants from Lucid Diagnostics, AbbVie, AMBU, Boston Scientific, Olympus, and ConMed. Dr Keihanian is a consultant for Lumendi, ConMed, and Neptune Medical. Dr Jawaid is a consultant for Creo Medical, Lumendi, and ConMed.

## Supplementary data

Video 1 Demonstration of a successful peroral endoscopic myotomy using a novel disposable scope platform.

